# The relationship between vitamin D status and depression in a tactical athlete population

**DOI:** 10.1186/s12970-019-0308-5

**Published:** 2019-09-10

**Authors:** Kelly A. Schaad, Asma S. Bukhari, Daniel I. Brooks, Justin D. Kocher, Nicholas D. Barringer

**Affiliations:** 1 U.S. Baylor Military Graduate Program in Nutrition, 3630 Stanley Road, San Antonio, TX 78234 USA; 2 Nutrition Services Department, Walter Reed National Military Medical Center, 8901 Wisconsin Avenue, Bethesda, MD 20889 USA; 3 Department of Research Programs, Walter Reed National Military Medical Center, 8901 Wisconsin Avenue, Bethesda, MD 20889 USA; 4 Health Readiness Center of Excellence, Capability Development Integration Directorate, 3630 Stanley Road, San Antonio, TX 78234 USA; 5 Military Nutrition Division, U.S. Army Research Institute of Environmental Medicine, 10 General Greene Ave, Natick, MA 01760 USA

**Keywords:** Vitamin D, Depression, Service members, Latitude, Vitamin D deficiency

## Abstract

**Background:**

Stressors inherent to the military, such as combat exposure, separation from family, and strenuous training, collectively contribute to compromised psychological resilience and greatly impact military performance.

**Methods:**

This retrospective review of records was conducted to determine whether vitamin D status was associated with diagnoses of depression and if diagnoses differed by geographic location.

**Results:**

Depression (defined using diagnostic codes) was more prevalent in individuals who were diagnosed with vitamin D deficiency (20.4%) than in individuals who were not (4.2%). After adjustment, vitamin D deficient diagnoses remained significantly associated with depression diagnoses (OR = 1.22; 95% CI, 1.11–1.33, *p* < 0.001). Furthermore, vitamin D deficient diagnoses were strongly associated with geographic latitude (*r*^*2*^ = 0.92, *p* = 0.002).

**Conclusion:**

These results suggest that service members stationed at installations located at northerly latitudes may be at increased risk for vitamin D deficiency. Furthermore, vitamin D deficient service members may be at higher risk for diagnosis of depression. As a number of military service members avoid reporting symptoms or seeking treatment, vitamin D status may be a useful screening tool to identify service members at risk for depression.

## Background

Depression is a serious, yet common, mental disorder with symptoms that may have detrimental effects on daily activities of living, especially in the work environment [1]. Development of depression is multifactorial and risk factors may include family history, major life changes, trauma, stress, physical illness, or the use of certain medications [1]. In the United States military, the prevalence of depression is further influenced by unique stressors such as participation in armed conflicts and extended separation from family [2]. Previous studies estimate that major depression is prevalent among 12% of currently deployed military personnel, over 13% of previously deployed military personnel, and almost 6% of never deployed military personnel [3].

Although a vast number of prevention, reintegration, and resiliency programs exist to promote mental health among service members, a 2014 Institute of Medicine (IOM) report concluded that many of these programs and screening tools are based on insufficient evidence and lack validated measures [4]. It is also suggested that service members feel uncomfortable reporting depression and are reluctant to seek medical care due to the belief that treatment will negatively impact their career path [5, 6]. Improved screening methods for mental disorders would ensure that military units maintain sufficient performance levels to quickly deploy in support of a full range of military operations and would assist service members with reintegration following deployment.

Vitamin D status, particularly vitamin D deficiency, may be a risk factor for the development of psychological disorders such as depression [7]. Eyles et al. [7] concluded that although observational in nature, there is a growing body of epidemiological research to support the association between vitamin D status and psychiatric outcomes. One proposed mechanism suggests that a deficiency of vitamin D alters the release of certain neurotransmitters in the brain [7, 8]. Although vitamin D is most commonly known for its role in calcium homeostasis and bone development, vitamin D receptors have been discovered in nearly all tissues in the body, indicating that vitamin D has additional physiological functions, including a neuroprotective role [9]. Vitamin D receptors expressed in neurotransmitter pathways can affect the synthesis of dopamine and serotonin, leading to a disruption in feelings of reward, motivation, pleasure, and drive [8, 9].

Vitamin D deficiency is common in the United States and is estimated to be prevalent among 35–41.5% of adults [10–12]. Few foods are naturally abundant in vitamin D and synthesis of the vitamin occurs primarily after exposure of the skin to ultraviolet (UV) radiation from sunlight. Optimal exposure to UV radiation occurs closest to the equator and during summer months, and is further influenced by elevation, season, cloud cover, and the time of day [13, 14]. Consequently, during winter months, vitamin D production is very low at latitudes above approximately 33° N or below 33° S [13]. Vitamin D deficiency is of interest within the military population, as Umhau et al. [15] and Funderburk et al. [16] reported that 30 and 21% of a sample of U.S. military service members were vitamin D deficient, respectively.

Previous studies assessing the relationship between vitamin D deficiency and depression show inconsistent results. In older adults, it is reported that lower vitamin D levels are associated with major depression, minor depression, and depressive symptoms [17, 18]. Likewise, Lapid et al. [19] found that in an elderly population, those with severe vitamin D deficiency were twice as likely to have a diagnosis of depression. However, in populations with comorbid conditions, such as multiple sclerosis, there was a negative correlation between vitamin D status and depression [20]. Additional factors may impact the relationship between vitamin D and depression, as Pan et al. [21] and Nanri et al. [22] both demonstrated that season and geographic location can affect the relationship between vitamin D and depression.

Existing studies on the relationship between vitamin D and depression have been conducted primarily in elderly populations or in populations with comorbidities and may not be applicable to active duty military personnel. Current demographics describe the U.S military population as relatively young, active, and with a low prevalence of chronic disease [23, 24]. Therefore, the purpose of this study was to assess the relationship between vitamin D status and depression in active duty service members. Furthermore, this study aimed to determine if there was a relationship between vitamin D status, depression, and geographic location. We hypothesized that diagnoses of vitamin D deficiency would be associated with diagnoses of depression. In addition, we hypothesized that there would be a higher number of vitamin D deficient diagnoses and depression diagnoses at military treatment facilities (MTFs) located above 33° N latitude than at military treatment facilities located below 33° N latitude.

## Methods

### Data source

Data for this study were obtained from the Military Health System (MHS) Data Repository (MDR), which contains information from a comprehensive network of military healthcare clinics that serve over 9.5 million beneficiaries [25]. Specifically, data were retrieved from the direct care component of the MDR. These records are referred to as direct care because the services are provided at MTFs operated solely by the Department of Defense (DoD) [25]. The data system, known as the Management and Analysis and Reporting Tool (M2), was used to query the MDR for Comprehensive Ambulatory Provider Encounter Records (CAPERs), which contain outpatient records for all encounters occurring at MTFs. Examples of outpatient encounters include primary care, wellness, specialty care, and group appointments. The M2 database was queried only for approved data and the workstation used for accessing data met DoD security requirements. This study was approved by the Human Protections Administrator at the Army Medical Department Center and School and the Institutional Review Board at Walter Reed National Military Medical Center.

### Extracted data

Study data were retrieved from the MHS for the years 2013 through 2015. Each record in the dataset contained: a pseudo identification number (an encrypted individual identifier that is comparable across datasets), the treatment facility name and identification, the common beneficiary category (confirmation that the individual was serving on active duty or active duty orders at the time of data retrieval), the specific beneficiary category (to separate Army Guard or Army Reserve on active duty orders from Regular Army status), gender (male or female), age group (four age categories: 18–24, 25–34, 35–44, and 45–64), sponsor rank group (the service members’ pay grade grouped in a broader category: enlisted junior, enlisted senior, officer junior, and officer senior), encounters (a count of records), and diagnosis (a diagnostic code for either vitamin D deficiency or depression as the chief complaint or as any of the secondary diagnoses up to the 10 maximum allowable codes).

### Exposure and outcome variables

The primary exposure variable, diagnosis of vitamin D deficiency, was defined as having at least one encounter with a primary or secondary Ninth Revision of the International Classification of Diseases, Clinical Modification (ICD-9-CM) code 268.9, Unspecified vitamin D deficiency [26]. The appearance of the diagnostic code for vitamin D deficiency in the record indicated that a clinical provider ordered a reliable laboratory assay to evaluate serum circulating levels of vitamin D prior to diagnosis. The Endocrine Society Clinical Practice Guidelines defines vitamin D deficiency as a 25-hydroxyvitamin D [25(OH)D] level of < 20 ng/mL (50 nmol/L) and recommends testing for the serum circulating levels of 25(OH)D in order to evaluate vitamin D status [13]. Although the Endocrine Society guidelines are commonly used for the testing and diagnosing of vitamin D status, due to limitations of using ICD-9-CM codes as the sole means to confirm diagnosis, this study was unable to verify if providers adhered to these methods.

The primary outcome variable, diagnosis of depression, was defined as having at least one encounter with either a primary or secondary diagnosis from the ICD-9-CM codes listed in Table 1 [26]. A diagnosis of depression is generally symptom-based for a specified period of time and determined using the diagnostic criteria outlined in the Diagnostic and Statistical Manual of Mental Disorders (DSM-V) [27]. The method for identifying diagnoses of depression by ICD-9-CM codes in this study is comparable to strategies used by previous studies [28, 29]. Similarly to the methods used for determining a diagnosis of vitamin D deficiency, the use of ICD-9-CM codes, to confirm diagnosis of depression, does not differentiate whether medical providers diagnosed accurately or within the accepted guidelines.

**Table 1:** Selected ICD-9-CM codes for depression and description

ICD-9-CM Code	Description
296.22	Major depressive affective disorder single episode
296.3	Major depressive affective disorder recurrent episode unspecified degree
296.31	Major depressive affective disorder recurrent
296.32	Major depressive affective disorder recurrent episode moderate degree
296.33	Major depressive affective disorder recurrent episode severe degree without psychotic behavior
296.9	Unspecified episodic mood disorder
311	Depressive disorder not elsewhere classified

### Study population

**Fig. 1:**
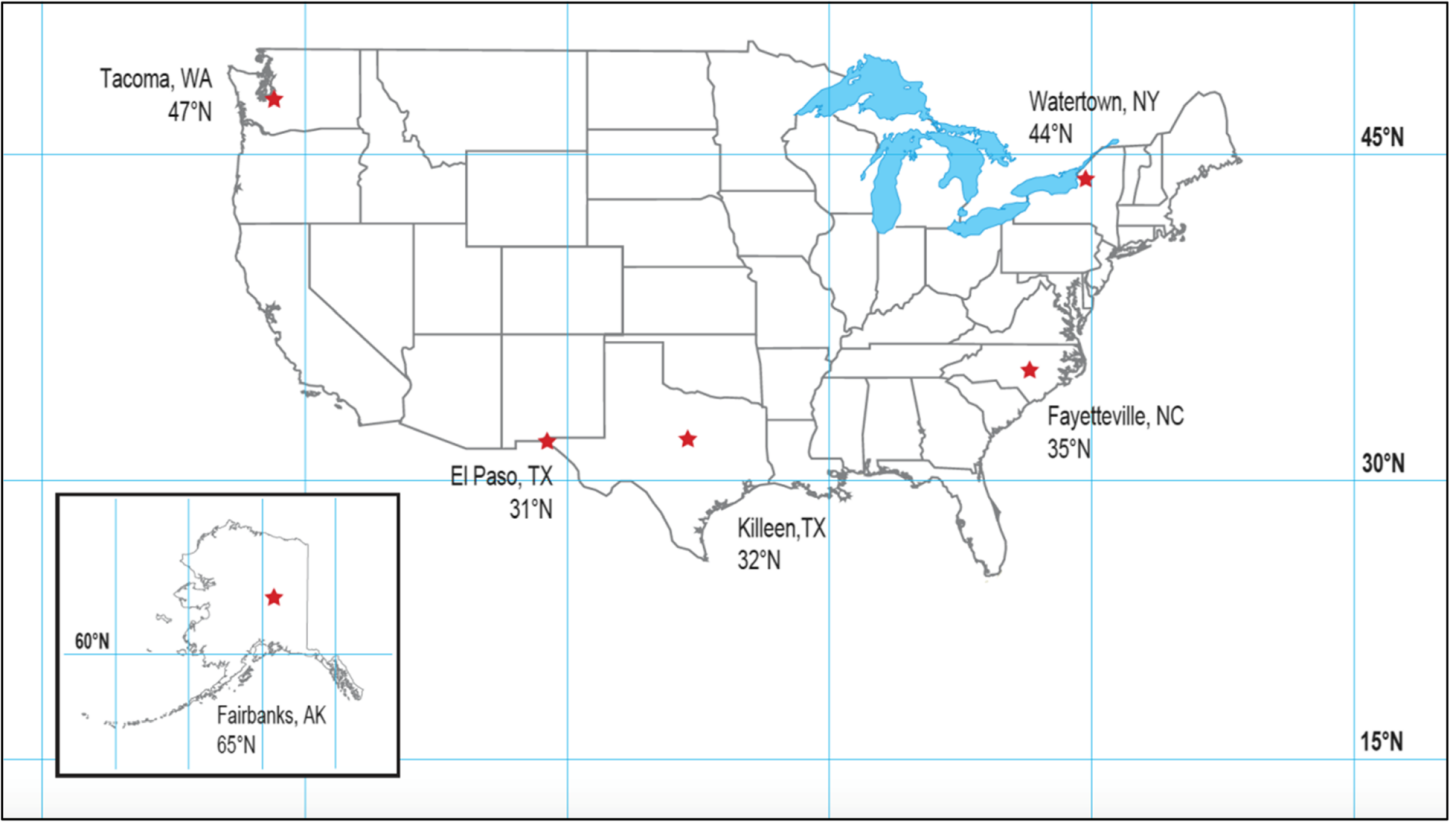
Geographic locations and associated latitudes for military treatment facilities where outpatient records were retrieved between the years 2013 and 2015

Individuals serving on active duty in the United States Army during the study period, with records for selected MTFs, were included for review (*n* = 483,683). Outpatient records were obtained for six military treatment facilities at varying geographic locations (Watertown, New York; Fairbanks, Alaska; Killeen, Texas; Tacoma, Washington; El Paso, Texas; and Fayetteville, North Carolina) in order to examine latitude as an independent variable (Fig. 1). Geographic location was a consideration in this study given that absorption of UV radiation, essential for vitamin D production, varies depending on degree of latitude. In order to capture personnel stationed at installations for a reasonable period of time, selection of MTFs on known training installations was avoided due to the transient nature of this population. Consequently, only MTFs located on installations without Initial Entry Training (IET) programs were included for review.

In order to associate demographic characteristics and medical outcomes without biasing the data, only one unique record was kept for each pseudo-identification number. Therefore, records with pseudo-identification numbers that appeared more than once (168,052) were reduced so that only the pseudo-identification number associated with the largest number of encounters was retained (*n* = 71,506), as a duplication indicated that an individual had outpatient encounters at multiple MTFs or had a change in demographic data during the study period. For records with multiple demographic characteristics but no maximum number of encounters, the first appearance in the dataset was kept arbitrarily (*n* = 6873). Records were eliminated to exclude individuals outside of the predetermined, 18–64 year old age parameter (99 total records excluded); to exclude military ranks other than junior enlisted, senior enlisted, junior officer, or senior officer (11,912 records excluded); and to exclude records without a listed gender (1 record excluded). Finally, individuals with ≥300 encounters were considered outliers and excluded from the dataset (189 total records excluded). After removing duplicates, outliers, and records excluded for the reasons above, 381,818 unique records remained for subsequent data analysis.

### Statistical methods

All demographic characteristics for this study were categorical and were described as a total number and frequency. A Chi-square test of independence was used to compare frequencies of vitamin D deficiency and depression between MTFs. The primary means of analysis was a binomial generalized linear model with a log link that was used to predict depression diagnosis. An initial model (Model 1) was created and included only vitamin D diagnosis; followed by a secondary model (Model 2) which included vitamin D diagnosis and a group of other covariates (MTF location, gender, age group, service type, career type, and career progression). Finally, a third model (Model 3) included these covariates as well as the number of associated encounters as a continuous variable. In this model, encounters were log transformed to approximate normality. Significance in each model was evaluated using a Type 3 Likelihood Ratio test. Odds ratios (and 95% confidence intervals) for individual parameters were extracted using exponentiated parameter estimates.

To examine the relationship between MTF latitude and vitamin D diagnosis, a separate model was built to predict vitamin D diagnosis (as in Model 3, above). From this model, the estimated rate of vitamin D deficiency at each MTF was calculated using least square means. These extracted rates were then correlated with the actual latitude of each MTF. Global alpha was set at *p* < 0.05 for each analysis and statistical analysis was conducted using IBM SPSS Statistics version 24.0; SPSS Inc., Chicago, Illinois and R Statistical Software (R Foundation for Statistical Computing, Vienna, Austria).

## Results

The demographic characteristics of the study sample, grouped by geographic location, are presented in Table 2. Of the total sample, 80.8% were active duty Army, 86.2% were male, and 86.3% were enlisted. The greatest number of records were obtained from the MTF located in Killeen, TX, whereas the fewest number of records were obtained from the MTF located in Fairbanks, AK. The largest age group was the 25–34 year old age group, followed by the 18–24 year old age group, which made up 40 and 36.9% of the sample, respectively. In total, 40% of personnel were senior ranking, compared to 60% of personnel that were junior ranking.

**Table 2:** Demographic characteristics of active duty service members by military treatment facility location

MTF Location	Fairbanks, AK		Watertown, NY		Tacoma, WA		Fayetteville, NC		El Paso, TX		Killeen, TX		Totals	
	*n* = 10,920	%	*n* = 31,130	%	*n* = 59,613	%	*n* = 88,826	%	*n* = 90,667	%	*n* = 100,662	%	*n* = 381,818	%
Characteristic														
Service Type														
Active Army	10,534	96.5	29,810	95.8	55,147	92.5	82,591	93	55,015	60.7	75,312	74.8	308,409	80.8
Active Guard	386	3.5	1320	4.2	4466	7.5	6235	7	33,652	39.3	25,350	25.2	73,409	19.2
Gender														
Male	9799	89.7	27,850	89.5	50,979	85.5	77,150	86.9	78,521	86.6	84,786	84.2	329,085	86.2
Female	1121	10.3	3280	10.5	8634	14.5	11,676	10.3	12,146	13.4	15,876	15.8	52,733	13.8
Age Group, Years														
18–24	5058	46.3	13,789	44.3	21,739	36.5	30,686	34.5	32,809	36.2	36,949	36.7	141,030	36.9
25–34	4243	38.9	11,762	37.8	24,689	41.4	37,917	42.7	34,431	38	39,504	39.2	152,546	40
35–44	1339	12.3	4479	14.4	10,103	16.9	15,325	17.3	16,338	18	17,606	14.5	65,190	17.1
45–64	280	2.6	1100	3.5	3082	5.2	4898	5.5	7089	7.8	6603	6.6	23,052	6
Career Type														
Enlisted	9846	90.2	27,504	88.4	51,312	86.1	75,154	84.6	77,752	85.8	87,890	87.3	329,458	86.3
Officer	1074	9.8	3626	11.6	8301	13.9	13,672	15.4	12,915	14.2	12,772	12.7	52,360	13.7
Career Progression														
Junior Rank	7571	69.3	20,735	66.6	37,483	62.9	49,434	55.7	54,132	59.7	59,806	59.4	229,161	60
Senior Rank	3349	30.7	10,395	33.4	22,130	37.1	39,392	44.3	36,535	40.3	40,856	40.6	152,657	40

Prevalence of vitamin D deficient diagnoses among this sample of active duty service members was < 1%, while the prevalence of depression was 4.37%. Diagnoses of depression were highest for the MTF located in Killeen, TX, followed by the MTF in Fairbanks, AK, and lowest for the MTF located in Watertown, NY. However, diagnoses of vitamin D deficiency were highest for the MTF located in Tacoma, WA, followed by the MTF for Fairbanks, AK, and lowest for the MTF located in Killeen TX. Raw frequencies and counts for each diagnosis, split by MTF location, are shown in Table 3.

**Table 3:** Vitamin D deficiency and depression diagnoses between the years 2013 and 2015 for active duty service members by military treatment facility location

MTF Location		Depression		Vitamin D	
	n	Count	Frequency	Count	Frequency
Watertown, NY	31,130	1008	3.24%	460	1.48%
Fairbanks, AK	10,920	474	4.34%	215	1.97%
Killeen, TX	100,662	6537	6.49%	310	0.31%
Tacoma, WA	59,613	2390	4.01%	1363	2.29%
El Paso, TX	90,667	2988	3.30%	410	0.45%
Fayetteville, NC	88,826	3291	3.70%	972	1.09%
Totals:	381,818	16,688		3730	

Overall, depression was more prevalent in individuals who were diagnosed with vitamin D deficiency (20.4%) than in individuals who were not (4.2%). Table 4 shows the results of predicting depression diagnosis using three separate models. Model 1 demonstrates that, without including other covariates, diagnoses of vitamin D deficiency were significantly associated with diagnoses of depression (OR = 5.84; 95% CI, 5.41–6.33 *p* < 0.001). This relationship remained significant (Model 2) after adjustment for socio-demographic characteristics and location (gender, age, service type, career type, career progression, and MTF location) (OR = 5.38; 95% CI, 4.95–5.88 *p* < 0.001).

**Table 4:** Unadjusted and adjusted odds ratio and level of significance for analysis of the association between diagnosis of vitamin D deficiency and diagnosis of depression

Parameter	Model 1^a^			Model 2^b^			Model 3^c^		
	OR	*p* value	CI	OR	*p* value	CI	OR	*p* value	CI
Vitamin D Status									
No Deficient Diagnosis	1			1			1		
Deficient Diagnosis	5.84	< 0.001	5.41–6.33	5.38	< 0.001	4.95–5.88	1.22	< 0.001	1.11–1.33
Gender									
Male				1			1		
Female				2.34	< 0.001	2.25–2.42	1.35	< 0.001	1.29–1.40
Age Group, Years									
18–24				2.22	< 0.001	2.05–2.40	1.13	0.006	1.04–1.24
25–34				1.55	< 0.001	1.45–1.67	1.15	< 0.001	1.07–1.25
35–44				1.17	< 0.001	1.09–1.23	1.14	0.001	1.05–1.23
45–64				1			1		
Service Type									
Active Guard				1			1		
Active Duty				3.29	< 0.001	3.11–3.48	1.24	< 0.001	1.16–1.32
Career Type									
Officer				1			1		
Enlisted				2.62	< 0.001	2.46–2.79	1.66	< 0.001	1.56–1.78
Career Progression									
Senior Rank				1			1		
Junior Rank				1.05	0.019	1.01–1.10	1.02	0.443	0.97–1.06
MTF Location									
Watertown, NY				1	< 0.001		1	< 0.001	
Fairbanks, AK				1.34	< 0.001	1.19–1.49	1.92	< 0.001	1.71–2.17
Killeen, TX				2.41	< 0.001	2.25–2.58	2.94	< 0.001	2.73–3.16
Tacoma, WA				1.19	< 0.001	1.10–1.28	1.53	< 0.001	1.41–1.66
El Paso, TX				1.35	< 0.001	1.25–1.45	1.99	< 0.001	1.85–2.16
Fayetteville, NC				1.15	< 0.001	1.07–1.24	1.54	< 0.001	1.43–1.67

*Abbreviations*: *OR* odds ratio, *CI* confidence interval

^a^includes only vitamin D diagnosis

^b^includes vitamin D diagnosis, MTF location, gender, age group, service type, career type, and career progression

^c^includes vitamin D diagnosis, MTF location, gender, age group, service type, career type, career progression, and associated encounters as a continuous variable

In an additional adjusted model (Model 3), the number of patient encounters, as a covariate, was also included for analysis. This variable, which represents the number of distinct medical encounters for each individual, was included after preliminary analysis indicated that it had a strong relationship with a diagnosis of both vitamin D deficiency and depression. Inclusion of the number of encounters as a covariate accounted for a large amount of the variance in depression diagnoses (OR = 3.66, CI, 3.59–3.72 *p* < 0.001) and strongly attenuated the relationship between Vitamin D Deficiency and depression (OR = 1.22; 95% CI, 1.11–1.33), however, this relationship remained significant (*p* < 0.001). Similarly, other covariates remained significant predictors for a diagnosis of depression, but with a reduced relationship.

**Fig. 2:**
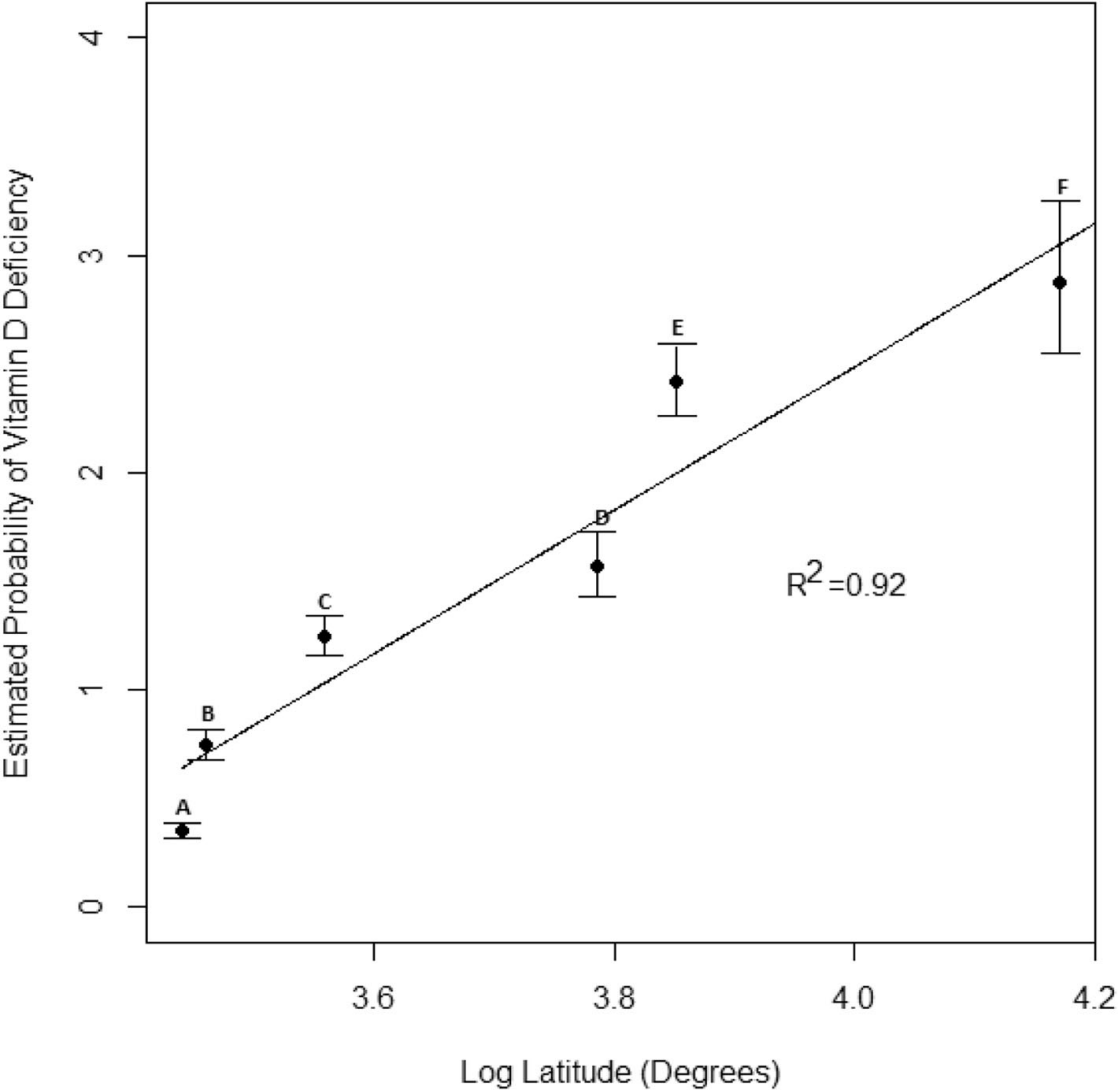
The relationship between vitamin D deficiency in active duty service members at six different MTF locations by degree of latitude

Finally, an analysis of the raw vitamin D deficiency percentages and (log transformed) latitude revealed a significant linear relationship (*r*^*2*^ = 0.75, *p* = 0.026). Rates of vitamin D deficiency were also modeled using a quasi-poisson generalized linear model, using depression, gender, age group, service type, career type, career progression, and encounters as covariates. Least squared mean predictions for each MTF, using an average number of encounters [13] and regular active duty Army status, were extracted from that model, which demonstrated an even stronger relationship between vitamin D deficiency and log latitude (Fig. 2) (*r*^*2*^ = 0.92, *p* = 0.002).

## Discussion

This retrospective cross-sectional study describes significant associations between vitamin D deficiency, depression, and geographic location. Of the 381,818 records that were included for analysis, a total of 3730 records contained a diagnostic code for vitamin D deficiency. The overall prevalence of vitamin D deficiency (< 1%), found in this study, is notably lower than previous studies of military personnel; which report that vitamin D deficiency ranges between 16 and 30% [15, 16]. The prevalence of vitamin D deficiency diagnoses were highest among males, the senior enlisted group, and the 25–34 year old age group. As this study only reviewed diagnoses, the actual number of service members who were examined for vitamin D status could not be determined. With this limitation, it is possible that a much larger number of personnel included in this study may have been vitamin D deficient but never received testing or diagnosis. Previous studies that report a higher prevalence of vitamin D deficiency in military populations describe research methods that include vitamin D testing or access to vitamin D status for the entire sample [15, 16]. Additionally, a portion of the low prevalence may be attributed to variation in testing and diagnostic methods. In this sample, vitamin D deficient diagnoses were obtained from several different MTFs which prevented the ability to control for variation in provider or facility policies. Although testing for vitamin D levels has become common practice in most healthcare facilities, potential variability in how providers identify patients as vitamin D deficient may lead to underreporting and misdiagnoses [30].

For depression diagnoses, 16,688 total records in the sample were found to contain one or more diagnostic code for depression. Although low, the overall prevalence of depression (4.37%) is comparable to that of previous studies which include military personnel. Diagnoses of depression were highest among females, the enlisted group, and the 18–24 year old age group. These results are not surprising as other studies have reported a higher prevalence of depression among females and in younger age demographics [3, 31]. Other studies estimate that the prevalence of depression in the U.S. military population varies widely (from 2 to 37.4%) [3]. Variability in the prevalence for depression in military samples may be related to differences in population samples, combat exposure, reporting methods, or the type of screening tool used by providers to diagnose for depression [5]. In previous evaluations of intervention programs for post-traumatic stress disorders (PTSD) and depression, it has been noted that missed diagnoses of depression may occur if providers complete the clinical assessment or referral process incorrectly [32, 33]. Additionally, as with the methods for determining vitamin D status, the nature of this study design prevented insight as to how many personnel in the sample were screened for depression. While questionnaire-based screening for depression occurs at set intervals in the military, it has been reported that in addition to errors in administration, a low prevalence of depression may also be related to the hesitance of military personnel to endorse symptoms during physical clinical assessments [32, 33].

The unadjusted relationship between vitamin D deficiency diagnoses and depression diagnoses, reported in this study, was much stronger (OR = 5.84) than the unadjusted relationship reported by similarly designed population-based studies [19, 34]. The relationship remained significant even after controlling for covariates known to be associated with depression such as location, gender, age, career type, career progression, and number of medical encounters (OR = 5.38). Personnel in the study sample without a diagnosis of vitamin D deficiency, potentially related to inconsistent screening of vitamin D status in the military, may indicate that there are actually a higher number of personnel with a depression diagnoses that are also vitamin D deficient. If this is the case, then the relationship between vitamin D deficiency and depression may be even stronger than reported in the present study. While limited by the observational nature of this study, the strong relationship between vitamin D deficiency and depression is of concern, as several other studies have demonstrated a causal relationship between vitamin D deficiency and depression. Both Vieth et al. [35] and Jorde et al. [36] reported that vitamin D supplementation improved participants’ well-being scores and depression scores, respectively. These findings may be of relevance to the military population as vitamin D deficiency is relatively easy to test for and may lead to earlier treatment or prevention of mental health conditions.

Though this study initially demonstrated a strong relationship between vitamin D and depression, the relationship was much weaker in the analysis which included the number of patient encounters as a covariate. After controlling for the number of encounters and demographic covariates, the adjusted relationship between vitamin D and depression (OR = 1.22) is very similar to the adjusted result (OR = 1.85) reported by Ganji et al. [34] As the design of this study cannot determine causality, it is unclear if individuals with depression have greater health care encounters as a result of diagnosis or if individuals with greater numbers of medical encounters are more likely to be diagnosed with depression. Beghofer et al. [37] also concluded that it was not possible to determine whether a diagnosis of depression is a risk factor for greater health care utilization, or instead an outcome of increased morbidity.

Despite a low overall prevalence of vitamin D deficiency, this study demonstrated a significant linear relationship (*r*^*2*^ = 0.92) between latitude and vitamin D deficiency percentages. This is particularly concerning as exposure to UV light is a critical source of vitamin D. Previous research also supports that differences in geographic location, even a one-degree difference in latitude, can have a significant impact on vitamin D status [38]. These results are meaningful, given that U.S. military personnel may be stationed at installations in geographic areas with inadequate UV exposure for several years. However, the analysis of these results is limited by the fact that this study could not control for other known covariates of vitamin D status such as skin tone, season of the year, weather, sunscreen use, body mass index (BMI), and vitamin D intake. Nevertheless, these findings further advocate for standardized vitamin D testing, especially for service members stationed at northern latitudes, as vitamin D deficiency may be related to depression.

Contrary to the relationship between vitamin D deficiency and latitude, this study did not find a significant relationship between depression and latitude. Rates of depression were highest for the MTF located in Killeen, TX and lowest for the MTF located in Watertown, NY. This may suggest that additional factors such as work-related stress, combat exposure, military occupational specialty, and the number of deployments, also affect the relationship, regardless of geographic location [39]. Additionally, other demographic factors, that are known to correlate with depression may be more strongly associated with depression than geographic location and were not controlled for in this study.

## Conclusions

Vitamin D deficiency is a worldwide problem with a growing number of known health implications. This study demonstrated that active duty service members with a diagnosis of vitamin D deficiency were much more likely to have a diagnosis of depression. Although further studies are needed to support this relationship, this may highlight the usefulness of vitamin D testing as an additional screening method for depression. Vitamin D testing, in conjunction with current screening tools, may be a better approach to circumvent service members who are reluctant to seek care for mental health conditions. This may especially be true for service members who are stationed at installation located in northern latitudes who present a higher risk for depression. Implementing standardized policies for vitamin D testing and diagnosing could be instrumental in increasing the likelihood that service members are diagnosed as vitamin D deficient and are identified for mental health interventions sooner rather than later.

## Data Availability

The datasets generated and analyzed during the current study are available from the corresponding author upon reasonable request.
